# Impact of Androgen Deprivation Therapy on Cognitive Function of Elderly Men With Prostate Cancer

**DOI:** 10.7759/cureus.69303

**Published:** 2024-09-12

**Authors:** Nourhan M Bassyouny, Mohamed M Gouda, Mai M Ezz El Din, Hala S Sweed, Rania M El Akkad

**Affiliations:** 1 Geriatrics, Faculty of Medicine, Ain Shams University Hospitals, Cairo, EGY; 2 Clinical Oncology, Faculty of Medicine, Ain Shams University Hospitals, Cairo, EGY

**Keywords:** androgen deprivation therapy, cognitive decline, elderly men, oncogeriatrics, prostate cancer

## Abstract

Background

Prostate cancer affects millions of men worldwide. Androgen deprivation therapy is the most prescribed medication for elderly men with prostatic cancer to slow and suppress the disease progression. Androgen deprivation therapy works on decreasing testosterone levels, and that can cause multiple side effects, including potential cognitive affection in the form of accelerating cognitive aging and potentially increasing the risk of dementia. This study is aimed at evaluating the impact of androgen deprivation therapy on the cognitive function of elderly men recently diagnosed with prostate cancer.

Methods

The current research is a prospective cohort study conducted on 85 elderly patients recently diagnosed with prostate cancer who are about to start androgen deprivation therapy within two weeks of the diagnosis. These patients were recruited from the oncology and geriatrics outpatient clinics of Ain Shams University hospitals and were followed up on androgen deprivation therapy for at least six months. Cognitive and depression assessments were done using the Montreal Cognitive Assessment Test and Montreal Cognitive Assessment Test-Basic (according to their education) and the Patient Health Questionnaire-9. The cases were assessed at the start, after two months, and after six months of androgen deprivation therapy use. Cognitively impaired or depressed patients were excluded at the beginning of the study.

Results

This study showed that 49 out of 85 (57.6%) of the studied participants had a lower Montreal cognitive assessment test score average after six months, indicating mild cognitive impairment. Cognitive domains such as visuospatial, language, and attention were affected. About one-third of the participants were diagnosed with depression after six months of the androgen deprivation therapy. All the depressed participants had cognitive impairment.

Conclusion

The use of androgen deprivation therapy carries the risk of cognitive decline and regression of some of the cognitive domains such as language, visuospatial, attention, and depression in the elderly with recently diagnosed prostate cancer who received ADT for six months. Conversely, depression could not be linked to cognitive decline. Further research should continue exploring the relationship between cognitive decline and ADT and seek strategies to mitigate these effects, ensuring comprehensive patient care targeting cognitive and psychological well-being.

## Introduction

Millions of men worldwide suffer from prostate cancer; over one million newly diagnosed cases can be attributed to prostate cancer. This disease accounts for 7% of newly diagnosed cancers in men worldwide (15% in industrialized nations), making it the second most frequent cancer in men after lung cancer. Prostate cancer is one of the main causes of cancer-related death in males, with more than 350,000 fatalities worldwide each year [1].

Generally, prostate adenocarcinoma cells are testosterone-sensitive [2]. Suppression of gonadal androgen production to castration levels results in prostate cancer cell death and clinical remission, which is evidenced by a drop in prostate-specific antigen (PSA) levels and/or radiographic tumor shrinkage [3].

Androgen deprivation therapy (ADT) is one option for treating prostate cancer. Conventional ADT includes luteinizing hormone-releasing hormone (LHRH) analogs, such as LHRH agonists (Goserelin, Leuprorelin, and Buserelin) and LHRH antagonists (Degarelix) or first-generation androgen-receptor signal inhibitors (ARSIs) (Bicalutamide and Flutamide). When LHRH agonists bind to the pituitary gland LHRH receptor, the pituitary overexcites and releases a burst of luteinizing hormone before ceasing the production of LH. LHRH antagonists, on the other hand, prohibit LHRH from binding to the pituitary receptor, directly inhibiting LH secretion and causing the cessation of testicular testosterone production and in medical castration [4].

As a result, ADT is used to treat around 50% of all cancer prostate patients. Most males are chemically castrated with LHRH agonists or antagonists, with or without anti-androgens [2]. These men typically receive ADT for the rest of their lives, which can slow the disease's progression. ADT can also enhance or prevent cancer-related symptoms like pain, pathologic fracture, or spinal cord compression secondary to bony metastatic disease in addition to extending median survival, limiting disability, and improving the patient's quality of life [5].

However, a variety of side effects may manifest during the use of this therapy, including cardiovascular disease, osteoporosis, anemia, hot flushes, metabolic diseases like insulin resistance, hyperlipoproteinemia, central adiposity, erectile dysfunction, muscle wasting, and gynecomastia [6].

Potential cognitive affection in patients receiving ADT has also received attention over the past 20 years. In the male brain, especially the areas involved in cognitive functions such as the thalamus, hippocampus, and cerebral cortex, there are receptors for free testosterone, particularly its more potent metabolite dihydrotestosterone, as well as estradiol (which is created when testosterone is converted into estradiol by the enzyme aromatase). These receptors work through a number of different pathways, including calcium channel activation, neurotransmitter modulation, and reduction in beta-amyloid production. When the levels of testosterone decline, a positive correlation between free testosterone levels and a decline in various cognitive abilities, including verbal memory, working memory, and visuospatial skills, was found [2].

These cognitive effects of ADT may help accelerate cognitive aging and possibly increase the risk of dementia in a population already at an increased risk [5]. Research employing objective methods to study cognitive functions has discovered cognitive deficits and an increased risk of developing dementia in men undergoing ADT [7]. Other studies, however, observed no change in cognitive functions with the long-term use of ADT [8]. Considering the fact that men over 65 account for half of all prostate cancer diagnoses, special consideration needs to be given [5]. The aim of this study is to evaluate the effect of androgen deprivation therapy on the cognitive function of elderly men with prostate cancer over time.

This article was previously presented as an abstract at the 44th Ain Shams University Medical Congress (ASUMC) on April 29, 2024.

## Materials and methods

This prospective cohort study was done on elderly male patients recently diagnosed with prostate cancer. They were recruited from Ain Shams University (ASU) outpatient oncology and geriatrics clinics. The timeframe was from September 2021 to December 2022.

This study was approved by the ethical committee of the faculty of medicine at ASU (FMASU MD 101/2021). An informed consent was obtained from each participant. Participants were oriented by the nature of the study, the test used, and the data extracted from this study. Confidentiality and privacy of data were ensured to all participants. The participation was voluntary, and the participants had the right to withdraw at any time.

Research participants

The study population included males aged 60 years and older, diagnosed with prostate cancer by history, examination, and investigations (labs, imaging, and biopsy results), and recently started ADT (within two weeks of diagnosis) and will continue on this treatment for at least six months. Exclusion criteria included: Patients who were known to have dementia or psychiatric disorders in their past medical history. Patients who had any past medical history of renal disease, hepatic disease, or cerebrovascular stroke. Patients who had cognitive impairment during their initial screening. Patients with depression, diagnosed at the start of the study, may cause pseudo-dementia (screened using the GDS 15 tool). Patients who were found to be delirious during any assessment. Patients who were receiving any medication that might affect their cognitive functions (sleep medications, psychotropic drugs, cognitive enhancer drugs). Severe hearing, visual, and functional impairments are preventing elderly patients from undergoing the assessment.

Data collection method

Participants initially collected at the start of the study were 93, but only 85 continued the six-month assessment period. Convenience samples were taken from the patients visiting the outpatient clinics.

Clinical assessment of the patients included personal history with special concern for age and education. The years of education were subdivided into illiterate, less than five years of education, more than five years of education, and college graduates. Past relevant medical history and drug history (including ADT therapy and its starting date) were collected.

Laboratory tests needed to stage prostate cancer included the PSA total levels [9]. The PSA level was obtained at the beginning of the study only. The pathology report was reviewed also for the type of cancer cells and the Gleason score [10]. The International Society of Urologic Pathologists (ISUP) employed the Gleason score to form the grade groups one to five. The imaging reviewed included an MRI pelvis and/or PET scan. These images were needed for the tumor node metastasis (TNM) staging system [11]. The PSA, Gleason score, and TNM staging system are used to classify and stage prostate cancer according to the European Association of Urology (EAU) risk classification [12].

Participants were interviewed about the other treatment options received for prostate cancer, such as surgical intervention, radiotherapy, or chemotherapy. Cognitive assessment was done on the participants using the Montreal Cognitive Assessment (MoCA) test [13] at baseline (within two weeks of starting ADT therapy), two months, and six months after the use of ADT. Changes in the collected data and scores were recorded and analyzed over time. The Arabic version of the MoCA test [14] was used for educated participants (participants who received more than five years of education and college graduates) to diagnose cognitive impairment. This test is designed as a rapid screening instrument for mild cognitive dysfunction. It assesses different cognitive domains. The total possible score is 30 points; a score of 26 or above is considered normal; a score of 18-25 points indicates mild cognitive impairment (MCI). A score of 10-17 points indicates moderate cognitive impairment. And fewer than 10 points indicate severe cognitive impairment. The test has a sensitivity of 90% and a specificity of 87% to detect MCI [13]. A certificate of training was achieved via the MoCA association badge number EGBASNO104918-01.

For participants who were illiterate or received less than five years of education, the Arabic version of the Montreal Cognitive Assessment-Basic (MoCA-B) was used [15]. MoCA-B is a modified version of the MoCA that is especially suitable for use in elderly subjects with a low level of education. The optimal cut-off score of <24/30 yielded 81% sensitivity and 86% specificity for MCI [16].

A depression assessment was done at the start and after six months of ADT use. Screening for depression was done using the Arabic version of the Patient Depression Questionnaire (PHQ-9). The PHQ-9 is a self-administered diagnostic instrument for depression, which scores each of the nine DSM-IV criteria as not at all=0, several days=1, more than half the days=2, and nearly every day=3. Add the numbers together to total the score. A PHQ-9 score total of 0 to 4 points equals “normal” or minimal depression. Scoring between 5 to 9 points indicates mild depression, 10-14 points indicates moderate depression, 15-19 points indicates moderately severe depression, and 20 or more points indicates severe depression. It can be used to make the diagnosis of depression and monitor the severity of depression and the patient’s response to treatment. The PHQ-9 has 61% sensitivity and 94% specificity to detect depression in adults [17].

The delirium screening tool was done using the Confusion Assessment Method (CAM) [18]. This test was done at the beginning of each interview to exclude patients with delirium at the time of assessment. The CAM demonstrated sensitivities from 94% to 100%, specificities from 90% to 95%, positive predictive accuracy of 91% to 94%, and negative predictive accuracy of 90% to 100% [19].

Statistical analysis

Our goal sample size is 73 cases to achieve the power of 0.80 to detect an effect size of at least 0.2 using one-way repeated measure ANOVA in three measurements with a correlation coefficient between measurements of 0.5 and non-sphericity correction of one. The sample should have an extra 20% to compensate for the dropouts, and the final sample size should be 85 cases. This sample produced a proportion of those with a decline of <1.5 standard deviations on two or more neuropsychological measures equal to 50% with a 95% confidence interval of 25% width.

The analysis of data was performed using SPSS Statistics version 16 (IBM Corp., Armonk, NY, USA). A description of all data in the form of the mean (M) and standard deviation (SD) for all quantitative variables was done. Frequency and percentage were calculated for all qualitative variables. Comparison between quantitative variables was done using the t-test to compare two groups. A comparison of qualitative variables was done using the chi-square test or Wilcoxon signed rank test when appropriate. A significant level was measured at p≤0.05 and highly significant at p≤0.01.

## Results

In our prospective study, 93 participants were initially recruited from the oncology and the geriatrics outpatient clinic, and only 85 continued the six-month assessment period. The participant’s ages ranged from 60 to 79 years, and the mean age was 67.83 ± 3.99. According to their level of education, they were subdivided into groups; 39 participants were college graduates (45.9%), and six participants received more than five years of education (7.1%) and had their cognitive abilities assessed using the MoCA test. Of the remaining 40 participants, 22 patients were illiterate (25.9%), and 18 patients received less than five years of education (21.1%), MoCA-basic was used to assess them (Table 1).

**Table 1:** Sociodemographic data and prostate cancer clinical characteristics and its relation to the MoCA test cut-off scores. ADT: Androgen deprivation therapy, EAU: European Association of Urology, SD: standard deviation, Sig.: significance, MoCA: Montreal Cognitive Assessment P-value > 0.05: Non-significant (NS) ; P-value < 0.05: Significant (S); P-value < 0.01: Highly significant (HS). *: Chi-square test.

		MoCA after 6 months		Test value	P-value	Sig.
		Normal	Abnormal			
		No. = 36	No. =49			
		N (%)	N (%)			
Level of education	Illiterate	8 (22.2)	14 (28.6)	1.889*	0.596	NS
	Less than five years	7 (19.4)	11 (22.4)			
	More than five years	4 (11.1)	2 (4.1)			
	College graduate	17 (47.2)	22 (44.9)			
Past medical history	Diabetes mellitus	21 (58.3)	30 (61.2)	0.072*	0.788	NS
	Hypertension	22 (61.1)	27 (55.1)	0.307*	0.580	NS
	Ischemic heart disease	16 (44.4)	25 (51.0)	0.359*	0.549	NS
Grade group (according to the Gleason score)	Grade 1	1 (2.8)	0 (0.0)	2.952*	0.399	NS
	Grade 2	9 (25.0)	19 (38.8)			
	Grade 3	16 (44.4)	18 (36.7)			
	Grade 4	10 (27.8)	12 (24.5)			
EAU risk groups	Intermediate risk	12 (46.2)	14 (53.8)	0.228*	0.892	NS
	High-risk	19 (40.4)	28 (59.6)			
	Metastatic	5 (41.7)	7 (58.3)			

The past medical history of the participants was reviewed and showed that 51 patients had diabetes mellitus (60.0%), 49 patients had hypertension (57.7%), and 41 patients had ischemic heart disease (48.2%) (Table 1). The patients were all diagnosed with adenocarcinoma and were subcategorized according to the EAU classification using TNM staging, PSA, and grade group into stages. According to the EAU risk groups, 26 patients (30.6%) had an intermediate risk, 47 patients (55.3%) had a high risk, and 12 patients (14.1%) were metastatic (Table 1). ADT was prescribed for at least six months. All the cases received 50 mg/day of Bicalutamide for two weeks, and then Goserelin (an LHRH agonist) was initiated.

All the participants received other modalities of treatment such as radiotherapy, radical prostatectomy, and chemotherapy (Docetaxel) according to the EAU guidelines. The participants had a negative CAM test before initiating the MoCA tests at each assessment.

The results of this study showed an affection for the MoCA test total score over six months. By the end of the study period, 49 out of 85 (57.6%) patients had an abnormal MoCA score (Table 2). The affection of the cognitive domains seen at the start of the study did not affect the total score, so these patients were not identified as cognitively impaired patients. 

**Table 2:** Montreal Cognitive Assessment (MoCA) test results for all participants over the six months period. MoCA-B: Montreal Cognitive Assessment-Basic, IQR: Interquartile range, Sig.: significance P-value > 0.05: Non-significant (NS); P-value < 0.05: Significant (S); P-value < 0.01: Highly significant (HS). *: Chi-square test; ≠: Wilcoxon signed rank test

MoCA test for educated group		At start, N (%)	After two months, N (%)	After six months, N (%)	Test value	P-value	Sig.
Visuospatial affection		5 (11.1)	0 (0.0)	6 (13.3)	6.136*	0.047	S
Language affection		0 (0.0)	0 (0.0)	22 (48.9)	52.566*	<0.001	HS
Attention affection		3 (6.7)	10 (22.2)	30 (66.7)	40.200*	<0.001	HS
Abstraction affection		0 (0.0)	0 (0.0)	0 (0.0)	–	–	–
Recall affection.		21 (46.7)	31 (68.9)	26 (57.8)	4.555*	0.103	NS
Orientation affection		13 (28.9)	19 (42.2)	19 (42.2)	2.269*	0.322	NS
Cut-off score interpretation	Normal	45 (100.0)	45 (100.0)	21 (46.7)	58.378*	<0.001	HS
	Abnormal	0 (0.0)	0 (0.0)	24 (53.3)			
Total score MoCA	Median (IQR)	29 (29 – 30)	29 (28 – 30)	25 (25 – 30)	4.666≠	<0.001	HS
	Range	28 – 30	27 – 30	24 – 30			
MoCA test for uneducated group		At start, N (%)	After two months, N (%)	After six months, N (%)	Test value	P-value	Sig.
Visuospatial affection		2 (5.0)	0 (0.0)	7 (17.5)	9.369*	0.009	HS
Language affection		2 (5.0)	2 (5.0)	26 (65.0)	51.200*	<0.001	HS
Attention affection		4 (10.0)	8 (20.0)	30 (75.0)	43.077*	<0.001	HS
Abstraction affection		0 (0.0)	0 (0.0)	0 (0.0)	–	–	–
Recall affection.		25 (62.5)	29 (72.5)	31 (77.5)	2.259*	0.323	NS
Orientation affection		15 (37.5)	16 (40.0)	19 (47.5)	0.891*	0.640	NS
Cut-off score interpretation.	Normal	40 (100.0)	40 (100.0)	15 (37.5)			
	Abnormal	0 (0.0)	0 (0.0)	25 (62.5)	63.158*	<0.001	HS
Total score MoCA-B	Median (IQR)	29 (29–30)	29 (28–29)	24 (24–28)	4.692≠	<0.001	HS
	Range	27–30	28–30	23–30			
Total score of MoCA and MoCA-basic		At start, N (%)	After two months, N (%)	After six months, N (%)	Test value	P-value	Sig.
Cut-off score interpretation	Normal	85 (100.0)	85 (100.0)	36 (42.4)	126.107*	<0.001	HS
	Abnormal	0 (0.0)	0 (0.0)	49 (57.6)			
Total score	Median (IQR)	29 (29–30)	29 (28–29)	25 (24–29)	58.968≠	<0.001	HS
	Range	27–30	27–30	23–30			

In the educated group (45 participants), after two months of ADT therapy, the total scores of the participants were within the normal reference ranges, but affection of attention, recall, and orientation started to show. After six months, 24 patients (53.3%) of the participating patients showed an abnormal MoCA test result, compared with the start of the study, which indicates mild cognitive impairment (MCI) according to the MoCA test reference ranges. The affection of cognitive domains such as language, attention, and visuospatial showed significance. While there were no significant changes over the follow-up period with abstraction, recall, and orientation (Table 2).

In the uneducated group (40 participants), 25 participants (62.5%) showed abnormal results after six months compared with the start of the study. Cognitive domains such as attention and recall started to be affected after two months of ADT therapy, but the patients’ final scores were within the normal range. After six months, the most affected cognitive domains were attention, visuospatial, and language, and showed statistical significance. While other cognitive domains assessed in the MoCA-Basic test were less affected, such as orientation, recall, and abstraction, and showed no statistical significance (Table 2).

The results of the MoCA test showed a non-significant relation with the education levels, past medical history, grade group, and EAU risk classification (Table 1). After six months, 23 participants had been positive for depression using the PHQ-9 test. Using the PHQ-9, 21 patients (91.4%) showed moderate depression, while only two patients (8.6%) showed mild depression, and none of the participants had moderately severe or severe depression.

All the patients who had a positive PHQ-9 test had MCI according to the MoCA score cut-off values. However, the lack of regression analysis was due to the absence of depressed participants with a normal MoCA or MoCA-Basic test (Table 3).

**Table 3:** PHQ-9 test results and its relation to the MoCA test cut-off values. MoCA: Montreal Cognitive Assessment, PHQ-9: Patient Depression Questionnaire, Sig.: significance P-value > 0.05: Non-significant (NS); P-value < 0.05: Significant (S); P-value < 0.01: Highly significant (HS). *: Chi-square test.

After six months		PHQ-9		Test value		P-value	Sig.
		Negative	Positive				
		N (%)	N (%)				
MoCA	Normal	36 (58.1)	0 (0.0)	23.167*	<0.001		HS
	Abnormal	26 (41.9)	23 (100.0)				

## Discussion

The prevalence of cognitive impairment increases with age. It was estimated that the increase is up to 25%-48% of community-living populations that are over 80 years of age [20]. Androgens have been reported to stimulate physical and cognitive ability, and long-term treatment with ADT might be associated with deterioration of physical ability, cognitive function, and quality of life [20].

The findings of this study align with a cohort study conducted on 154,089 patients with prostate cancer, which has provided valuable insights. Notably, this study has the longest mean follow-up period reported to date, spanning over eight years. The findings of this study indicate a significant association between Alzheimer's dementia (AD) and dementia, as evidenced by hazard ratios of 1.140 and 1.20, respectively. The observed decline in cognitive function can be attributed to the extended follow-up period and the varying prescription of ADT regimens. It is important to note that the participants were assessed within two years of initiating ADT using different cognitive assessment tools. Consequently, the likelihood of detecting MCI was relatively low [21].

Gunlusoy et al. [22] conducted a study with two groups of 78 participants each. Group 1 received 12 months of continuous complete ADT for prostate cancer, while group 2 underwent radical prostatectomy without additional treatment. Cognitive function was assessed using the MoCA test. Results showed that group 1 had lower mean total scores compared to group 2, particularly in language ability and short-term memory capacity (P < 0.05). However, there were no significant differences in attention, executive functions, visuospatial abilities, abstract thinking, calculating abilities, or orientation. The discrepancy in the affection of attention and visuospatial affection compared to our study can be attributed to the longer study duration and the presence of a control group to compare the results [22].

Another study demonstrated that patients with ADT exhibit significant cognitive impairment, including language ability, short-term memory, prospective memory, mental flexibility, inhibitory control, and emotional psychology, compared to those without ADT. Cognitive dysfunction typically occurs within six to 12 months after ADT treatment [23].

Out of 85 participants, 23 patients tested positive for depression using the PHQ-9 test interview. Most of the patients showed moderate depression within six months of ADT therapy.

Our results are consistent with Ceylan et al. [24] conducted a prospective, comparative study to explore the association between ADT and depression, as well as the impact of depression on cognitive functions in men with locally advanced or metastatic prostate cancer. The study included a total of 144 prostate cancer patients, with 72 patients in group 1 receiving continuous ADT treatment for 12 months and 72 patients in group 2 (the control group) undergoing radical prostatectomy without additional treatment. To assess the effects of ADT on depression and cognitive functions, the researchers utilized the MoCA and Hamilton Depression Rating Scale (HAM-D) tests. The post-treatment results of the MoCA test revealed that both groups of patients had lower mean total scores, particularly in the areas of language ability and short-term memory capacity. When comparing the two groups using the HAM-D tests, significantly higher scores were observed in group 1. Furthermore, a relationship was found between depression and the deterioration of language, attention, and memory functions in the sixth and 12th months [24].

In a recent retrospective cohort study utilizing the TRICARE Military Database, researchers examined the impact of ADT on the risk of depression and dementia in a group of 9,117 men aged 40-64 who had been diagnosed with localized prostate cancer. The study revealed that patients receiving ADT had a significantly higher risk of developing depression and dementia [25]. This study followed patients from the initiation of ADT until their death, last encounter, or the end of the study period. So, the longer follow-up period might have detected the development of dementia rather than just MCI, which was detected by our study due to a shorter follow-up period.

Lastly, a meta-analysis involving 168,756 individuals also examined the association between ADT use and depression. The findings indicated that ADT use was associated with a 41% increased risk of depression. And depression was linked to a higher risk of cognitive dysfunction [26].

In contrast to our results, a study was conducted in 2021 to examine the effects of androgen deprivation therapy on cognitive function and depressive symptoms in men with an average age of 70.8 years. The participants were assessed at two timepoints: six months into treatment and 12 months later. The analysis of cognitive function using the Mini-Mental State Examination (MMSE) scores showed a significant increase after one year of treatment. However, when using the Brief Scale for Cognitive Evaluation (BCog) scores, no significant changes in cognitive function were observed before and after one year of treatment [27]. On the other hand, the analysis of depressive symptoms using the Geriatric Depression Scale showed significant changes after one year of treatment. Men reported experiencing more depressive symptoms following the treatment. There were no statistically significant differences in cognitive performance between men with depressive symptoms and those without [27].

Another study found contradictory results to our study. They compared the cognitive function of 70 men with prostate cancer who received ADT to two other groups: non-ADT-treated men and non-prostate cancer controls. The study found that the prevalence of cognitive impairment was low (4%-13%) and there were no significant differences between the groups [28]. However, a study in the UK was done on 15,310 versus 15,593 prostate cancer patients with or without ADT, respectively. This study utilized the National Clinical Practice Research Datalink and showed that ADT did not increase the risk of dementia during a mean follow-up period of more than four years [28].

The ability to draw consistent conclusions on this matter is complicated due to the diversity in study designs, methodologies, types of ADT agents used, treatment duration, the timing to start the follow-up period after ADT initiation, and different cognitive tests used. Also, variations in the participant’s characteristics across different studies, as regards their age, education, family history of dementia, smoking, alcohol consumption, physical activity, nutrition, and frailty, make it challenging to definitively conclude whether ADT directly causes cognitive dysfunction or not. Until a clearer understanding of the association between ADT and cognitive dysfunction, healthcare professionals must inform patients who are about to undergo ADT about the potential cognitive effects, depression, and functional decline and provide them with resources for maintaining good brain and physical health.

Limitations and strengths

This study is conducted in a single-center study; it may not be representative of all the geriatric population with prostate cancer in Egypt. A longer study period is needed to further assess either the progression or the reversibility of the MCI developed by prostate cancer patients on ADT therapy. A larger sample size will help in better identification of each significant predictor of cognitive impairment on its own and create a better regression model. Nevertheless, our study highlights the need for routine cognitive and depression assessment inclusion in the follow-up of elderly with prostate cancer on hormonal therapy to ensure early detection, interference, and improvement of the quality of life.

## Conclusions

The use of ADT carries the risk of cognitive decline, and regression of some of the cognitive domains such as language, visuospatial, attention, and depression in the elderly with recently diagnosed prostate cancer who received ADT for six months. Conversely, depression could not be linked to cognitive decline. Further research should continue to explore the relationship between cognitive decline and ADT and seek strategies to mitigate these effects, ensuring comprehensive patient care targeting both cognitive and psychological well-being.
